# Expression and Significance of the HIP/PAP and RegIIIγ Antimicrobial Peptides during Mammalian Urinary Tract Infection

**DOI:** 10.1371/journal.pone.0144024

**Published:** 2015-12-10

**Authors:** John David Spencer, Ashley R. Jackson, Birong Li, Christina B. Ching, Martin Vonau, Robert S. Easterling, Andrew L. Schwaderer, Kirk M. McHugh, Brian Becknell

**Affiliations:** 1 Division of Nephrology, Department of Pediatrics, The Ohio State University College of Medicine, Columbus, Ohio, United States of America; 2 Center for Clinical and Translational Research, Research Institute at Nationwide Children’s, Columbus, Ohio, United States of America; 3 Biomedical Sciences Graduate Program, The Ohio State University College of Medicine, Columbus, Ohio, United States of America; 4 Division of Urology, Department of Surgery, The Ohio State University College of Medicine, Columbus, Ohio, United States of America; 5 Department of Pediatrics and Internal Medicine, The Ohio State University College of Medicine, Columbus, Ohio, United States of America; 6 Medical College of Ohio, Toledo, Ohio, United States of America; 7 Department of Anatomy, The Ohio State University College of Medicine, Columbus, Ohio, United States of America; 8 Center for Molecular and Human Genetics, Research Institute at Nationwide Children’s, Columbus, Ohio, United States of America; Cedars-Sinai Medical Center, UNITED STATES

## Abstract

Recent evidence indicates that antimicrobial peptides (AMPs) serve key roles in defending the urinary tract against invading uropathogens. To date, the individual contribution of AMPs to urinary tract host defense is not well defined. In this study, we identified *Regenerating islet-derived 3 gamma* (RegIIIγ) as the most transcriptionally up-regulated AMP in murine bladder transcriptomes following uropathogenic *Escherichia coli* (UPEC) infection. We confirmed induction of RegIIIγ mRNA during cystitis and pyelonephritis by quantitative RT-PCR. Immunoblotting demonstrates increased bladder and urinary RegIIIγ protein levels following UPEC infection. Immunostaining localizes RegIIIγ protein to urothelial cells of infected bladders and kidneys. Human patients with UTI have increased urine concentrations of the orthologous Hepatocarcinoma-Intestine-Pancreas / Pancreatitis Associated Protein (HIP/PAP) compared to healthy controls. Recombinant RegIIIγ protein does not demonstrate bactericidal activity toward UPEC *in vitro*, but does kill *Staphylococcus saprophyticus* in a dose-dependent manner. Kidney and bladder tissue from RegIIIγ knockout mice and wild-type mice contain comparable bacterial burden following UPEC and Gram-positive UTI. Our results demonstrate that RegIIIγ and HIP/PAP expression is induced during human and murine UTI. However, their specific function in the urinary tract remains uncertain.

## Introduction

The human urinary tract ranks among the most common sites of bacterial infections.[1] Over 50% of women experience at least one case of urinary tract infection (UTI) in their lifetime.[2] While most UTI localize to the bladder (cystitis), ascending bacteria can invade the renal parenchyma, resulting in acute pyelonephritis (APN). In 2012, APN led to 200,000 emergency department visits, 64,000 hospital admissions, and $378 million in inpatient hospital costs in the United States alone.[3] The frequency and severity of UTI increase in patients with urinary tract obstruction, cystic kidney disease, neurogenic bladder, kidney stones, diabetes mellitus, and recipients of kidney transplants.[4] Treatment for recurrent UTI exposes patients to repeated courses of antibiotics, which promotes the emergence of antibiotic-resistant pathogens and restricts the physician’s treatment options.[5] Altogether, this mandates a greater understanding of UTI pathogenesis in order to thwart invading bacteria and protect the urinary tract.

The innate immune response is responsible for swift and efficient eradication of pathogens, as well as triggering the adaptive immune response. In the urinary tract, invading bacteria face defenses consisting of (1) bladder emptying and mucous production; (2) exfoliation and regeneration of the urothelial epithelium; (3) recruitment of phagocytes that possess bactericidal activity and engulf microbes; (4) cytokines and chemokines; (5) and AMPs.[6,7,8,9,10] Recent evidence suggests that AMPs protect the urothelium from invading pathogens. Urothelial cells or invading leukocytes produce AMPs, like α- and β-defensins, cathelicidin, and ribonuclease 6 and 7, that directly kill uropathogens. Other urinary tract proteins, including lipocalin and lactoferrin, suppress bacterial growth by scavenging key micronutrients.[6,7,8,9]

The goal of this study was to identify AMPs induced during experimental UTI using an unbiased global transcriptome profiling approach. In doing so, we identified *Regenerating islet-derived 3 gamma* (RegIIIγ) as the most transcriptionally induced AMP in mouse bladders. RegIIIγ, which was initially identified in Paneth cells of the small intestine, is a secreted lectin that exerts antimicrobial activity against Gram-positive bacteria. Prior studies implicate RegIIIγ as a mediator of mucosal immunity in other organ systems. [11,12,13,14,15,16,17] Therefore, in this study we localized RegIIIγ urinary tract expression and assessed its role in uropathogen clearance.

## Methods

### Bacterial strains

UTI89 is a type I-piliated UPEC strain isolated from a patient with cystitis.[18] CFT073 is a UPEC strain isolated from the blood and urine of a patient with APN.[19] We also used Gram-positive human urinary tract isolates *Enterococcus faecalis* 0852 and *Staphylococcus saprophyticus* 7108. [20] [21,22]

### UTI model

Use of the RegIIIγ^*-/-*^ and WT mice for experimental UTI model was approved by The Research Institute at Nationwide Children’s Hospital Institutional Laboratory Animal Care and Use Committee (Welfare Assurance Number A3544-01), protocol AR13-00057 (BB). Animals were euthanized by cervical dislocation under deep isoflurane anesthesia. Internal organs were removed to ensure demise. *RegIIIγ* ^*-/-*^ mice were generously provided by Dr. Lora Hooper (UT Southwestern) and backcrossed at least 7 generations to C57BL/6 prior to experiments.[15] *RegIIIγ* ^*-/-*^ or C57BL/6 (wild-type (WT)) females were generated by homozygous breeding and maintained on standard chow (Harlan, Indianapolis, IN) with 12 hour light/dark cycles. Animals were housed in ventilated cages and transferred to a Biosafety Level 2 room one day prior to inoculation. Uropathogenic bacteria were inoculated from glycerol stocks into LB medium and expanded statically for 16 hours at 37°C. Experimental UTI, tissue isolation, and assessment of bacterial burden were performed as previously described.[23] The bacterial inoculum was 10^8^ colony forming units (CFU) in 50 μl except where otherwise indicated.

### RNA extraction and microarray hybridization

Total RNA was extracted using the mirVana^TM^ kit (Life Technologies, ThermoFisher Scientific). Tissue disruption was accomplished with a TissueLyser II instrument (Qiagen, Carlsbad, CA). Sample concentration was determined using the NanoDrop® ND-1000, and RNA integrity was analyzed using the Agilent 2100 Bioanalyzer Lab-On-A-Chip Agilent 6000 Series II chip. Sample labeling and hybridization was performed per the manufacturer’s protocol. Samples were hybridized to the SurePrint G3 Mouse GE 8x60K Microarray and scanned using the Agilent G2505C Microarray Scanner. Raw data were quality-assessed, filtered for outliers and normalized to remove non-biological variation. A 10% false discovery rate and p-value adjustment using the Bioconductorlimma package yielded significantly differentially expressed genes (Core Facility, Nationwide Children’s, Columbus, OH). Microarray data may be accessed at Gene Expression Omnibus (www.ncbi.nlm.nih.gov/GEO), using accession number GSE72007.

### QRT-PCR

Up to 2 μg of total RNA were reverse transcribed using random hexamer oligonucleotides in a 20 μl reaction volume (Verso cDNA Synthesis Kit, Thermo Scientific, Waltham, MA). After dilution to 60 μl with sterile water, 1 μl cDNA was used as template in a quantitative (q)RT-PCR reaction. Duplicate PCR reactions were performed using 2x master mix (Fisher). VIC-MGB labeled *Gapdh* and FAM-MGB labeled *RegIIIγ* or *RegIIIβ* primer/probe sets were used in separate reactions (Applied Biosystems, Carlsbad, CA). Alternatively, PCR reactions were performed using 2x master mix containing Sybr Green (Fisher) and 1 pmol of gene-specific primer pairs (**S1 Table**). PCR products were amplified and detected using the 7500 Real-Time PCR System (Applied Biosystems). PCR threshold cycles (CT) were determined, and each cDNA was normalized for *Gapdh* content (ΔCT). Relative expression changes were calculated using the 2^^-ΔΔCT^ method, normalizing to a common uninfected kidney or bladder cDNA pool.[24]

### Immunoblotting and immunostaining

Western blot analysis and immunostaining were performed as previously described.[23,25] Primary antibody used for Western blot analysis included: rabbit anti-RegIIIγ antibody, 1:1000, Dr. Lora Hooper, UT Southwestern, Dallas, TX; mouse anti-Gapdh antibody, MAB374, Chemicon, 1:5000. For immunofluoresence (IF), the RegIIIγ antibody was diluted 1:400, then detected with AlexaFluor 488-conjugated donkey anti-rabbit secondary antibody (Jackson Immunoresearch, West Grove, PA). Negative controls sections were incubated with rabbit serum in place of RegIIIγ antisera.

### Study approval and human urine specimens

Informed written consent was obtained from all patients participating in this study. For subjects less than 18 year of age, written parental/guardian consent was obtained. The Nationwide Children’s Hospital (NCH) Institutional Review Board approved this study along with the consent process and documents (IRB07-00383).[26] Non-infected and infected urines samples were obtained from children presenting to the NCH emergency department or the nephrology clinic. The diagnosis of a UTI was made by a positive urine culture according to the American Academy of Pediatrics Guidelines.[27] All infected urine samples had >10^5^ CFU/mL *E*. *coli*.

### Human urinary HIP/PAP levels

Urinary HIP/PAP (PAP) concentrations were measured by enzyme linked immunosorbent assay (ELISA; Dynabio, Marseille, France). Results were normalized to urine creatinine (Cr) as measured by ELISA (Oxford Biomedical Research, Rochester Hills, MI).

*Amino acid alignments*: Full-length Human HIP/PAP (accession number NP_620355.1) and mouse RegIIIγ (NP_035390.1) amino acid sequences were obtained from the National Center for Biotechnology Information (www.ncbi.nlm.nih.gov/protein) and aligned using MegAlign (DNAStar, Madison, WI) with the Clustal W method.

### Bactericidal activity of recombinant RegIIIγ

Recombinant, N-terminally processed RegIIIγ was purified from *E*. *coli* inclusion bodies as described.[28,29] UPEC strain UTI89 (10^5^ CFU) was incubated with 0–10 μM RegIIIγ for 2 hours at 37°C in 10 mM MES, pH 5.5 with 25 mM NaCl. Samples were then serially diluted, plated overnight on LB, and colonies were enumerated.

### Statistics

For comparing bacterial burden in WT versus *RegIIIγ* ^*-/-*^ mice, geometric means of log-transformed CFUs from kidneys and bladders were compared by the Mann-Whitney U test (GraphPad Software, La Jolla, CA). Similarly, for comparison of fold-differences in mRNA expression, Mann-Whitney U test was used. *P* values of < 0.05 were considered significant.

## Results

### Global analysis of AMP mRNA expression in experimental cystitis

To identify AMP undergoing transcriptional up-regulation by UPEC in an unbiased manner, we evaluated bladder transcriptomes at baseline and following experimental UTI by oligonucleotide microarray. We performed experimental cystitis in C57BL/6J female mice, and compared transcriptomes from uninfected bladders versus bladders harvested 2, 6, and 48 hours following intravesical inoculation with 10^8^ CFU of UPEC strain UTI89. We interrogated these data using 98 *Mus musculus* gene symbols encoding known or putative AMPs (**S2 Table**)[6,30,31] and identified 7 AMP transcripts with ≥ 4-fold increase in expression during experimental cystitis (**Table 1**). These genes encode AMPs with varying mechanisms of antimicrobial activity including bactericidal activity (*RNase6*, *RegIIIγ*) as well as metal sequestration (*S100a8*, *S100a9*, *Lcn2*, *Ltf*).[6,13]

**Table 1:** Regulation of AMP mRNA expression during experimental cystitis. Genes encoding known or putative AMP with ≥ 4-fold change (FC) are depicted. False discovery rate (FDR) < 10 is considered significant; n.s. = not significant.

	2 HPI		6 HPI		48 HPI	
AMP	FC	FDR	FC	FDR	FC	FDR
*Reg3γ*	15.5	2.2	167.4	1.3	90.1	1.7
*S100a9*	5.3	4.1	59.4	1.3	14.5	7.6
*S100a8*	4.0	3.3	39.3	1.3	10.4	7.3
*Lcn2*	n.s.	n.s.	9.1	1.3	4.9	8.7
*Ltf*	n.s.	n.s.	5.1	1.3	12.5	3.2
*Reg3β*	n.s.	n.s.	4.3	1.3	n.s.	n.s.
*RNase6*	n.s.	n.s.	n.s.	n.s.	4.3	2.1

**Fig 1:**
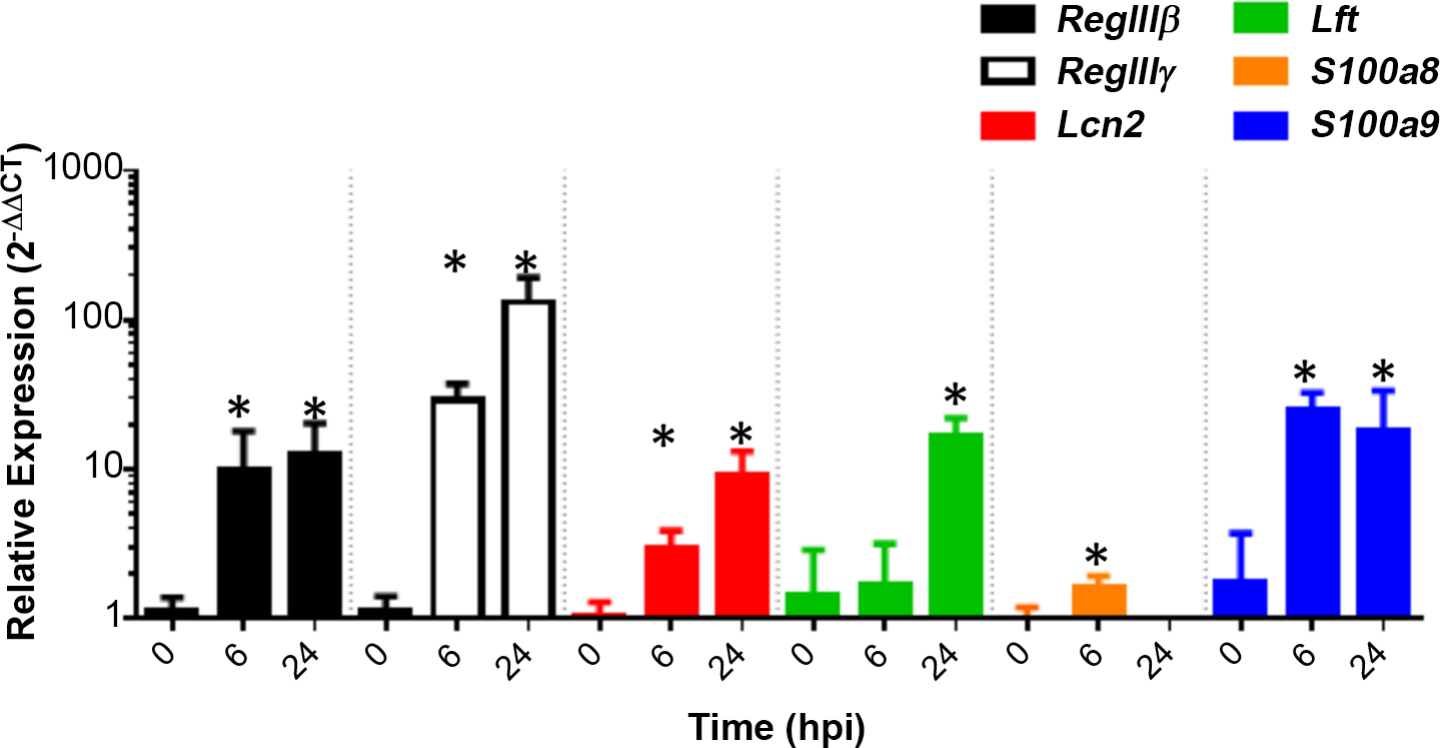
Confirmation of AMP mRNA regulation during experimental cystitis by qRT-PCR. The fold-change in AMP mRNA expression 6 and 24 hpi, relative to a pool of uninfected bladders, is shown. * *P* < 0.05, Mann-Whitney U test versus uninfected bladders (n = 4 bladders/group, uninfected and 6 hpi; n = 5 bladders/group, 24 hpi).

Previously, we demonstrated that significant *RNase6* mRNA induction occurs 48 hours post infection (hpi) with UPEC.[23] Therefore, we evaluated expression of the remaining 6 AMPs induced during UTI by quantitative reverse transcription polymerase chain reaction (qRT-PCR) at 6 and 24 hpi (**Fig 1**). QRT-PCR confirmed significant induction of *RegIIIβ*, *RegIIIγ*, *Lcn2*, *Ltf*, *S100a8*, and *S100a9* mRNA levels following infection (**Fig 1**). Our results also identify *RegIIIγ* as the AMP with the highest fold-induction following UPEC challenge.

### RegIIIγ mRNA and protein expression during cystitis

**Fig 2:**
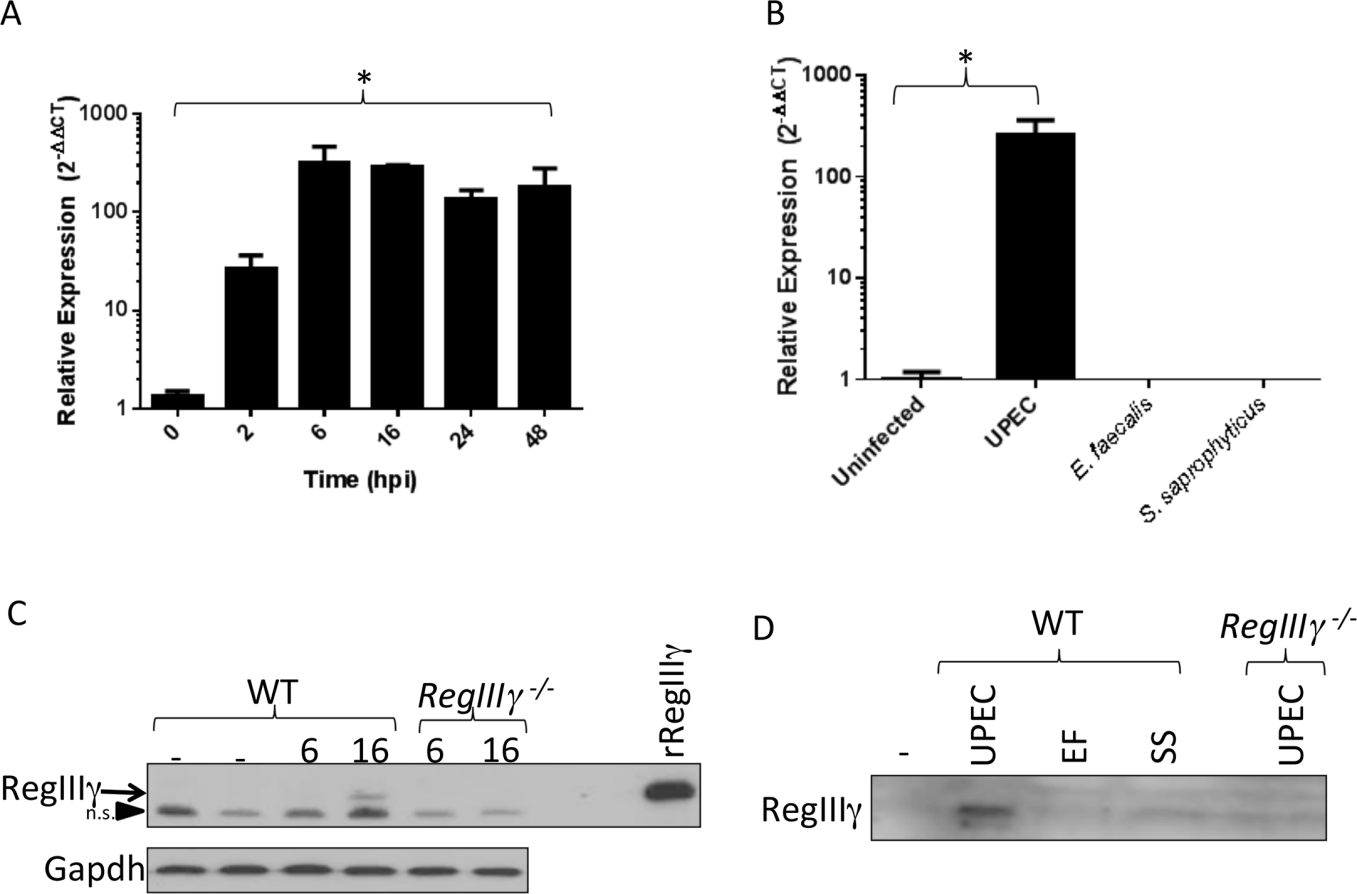
*RegIIIγ* mRNA and RegIIIγ protein expression are induced during UPEC cystitis, and RegIIIγ is secreted into the urinary stream following UPEC infection. (**A**) Fold-change in bladder *RegIIIγ* mRNA levels in response to intravesical inoculation of 10^8^ CFU of UPEC strain UTI89. * *P* = 0.0286, Mann-Whitney U test versus uninfected bladders (n = 4 bladders/group). (**B**) Relative *RegIIIγ* mRNA levels increase 24 hpi following inoculation with 10^8^ CFU of UPEC, but not in response to Gram-positive uropathogens. * *P* = 0.0286, Mann-Whitney U test, UPEC versus uninfected bladders (n = 4 bladders/group). (**C**) RegIIIγ protein (arrow) is induced in wild-type (WT) bladder 16 hpi with UPEC, but expression is absent at earlier times, and as well as in *RegIIIγ* ^-/-^ bladders. Bladder RegIIIγ co-migrates with recombinant (r) RegIIIγ protein. A non-specific reactive band (n.s., arrowhead) migrates more rapidly and is present in WT and *RegIIIγ* ^*-/-*^ bladder lysates. (**D**) RegIIIγ protein is undetectable in sterile WT urine, but secreted 24 hpi with UPEC. Gram-positive inocula, *E*. *faecalis* (EF) and *S*. *saprophyticus* (SS), fail to induce RegIIIγ secretion, and RegIIIγ immunoreactivity is absent in *RegIIIγ* ^*-/-*^ urine 24 hpi with UPEC.

In non-infected urinary tract tissues, qRT-PCR demonstrates significantly higher expression of *RegIIIγ* mRNA in bladder compared to ureter and kidney (*p* = 0.0286, Mann-Whitney U test)(**S1 Fig**). To completely characterize *RegIIIγ* mRNA expression during experimental UTI, we profiled *RegIIIγ* mRNA expression over a more extensive time course. As predicted by our microarray experiment, *RegIIIγ* mRNA levels significantly increase as early as 2 hpi and peak 16 hpi with 300-fold higher expression over uninfected bladders (*p* = 0.0286, Mann-Whitney U test)(**Fig 2A**). The induction of *RegIIIγ* mRNA was specific to UPEC, as intravesical injection of PBS carrier or inoculation of the Gram-positive uropathogens, *Enterococcus faecalis* and *Staphylococcus saprophyticus*, did not elicit a significant expression change (**Fig 2B**).

To determine if RegIIIγ protein expression parallels *RegIIIγ* mRNA expression, we performed Western blot analysis. Following UPEC inoculation, we detected RegIIIγ protein by 16 hpi (**Fig 2C**) and as late as 72 hpi (data not shown). In addition, we identified RegIIIγ protein in urine of mice 24 hpi with UPEC, but not in sterile urine or following intravesical administration of Gram-positive uropathogens (**Fig 2D**). In urine, we visualized a more rapidly migrating RegIIIγ-immunoreactive band, consistent with reports that secreted RegIIIγ undergoes proteolytic processing.[12,29] RegIIIγ protein was not detected in *RegIIIγ* ^-/-^ bladders or urine following UPEC inoculation (**Fig 2C and 2D**).

**Fig 3:**
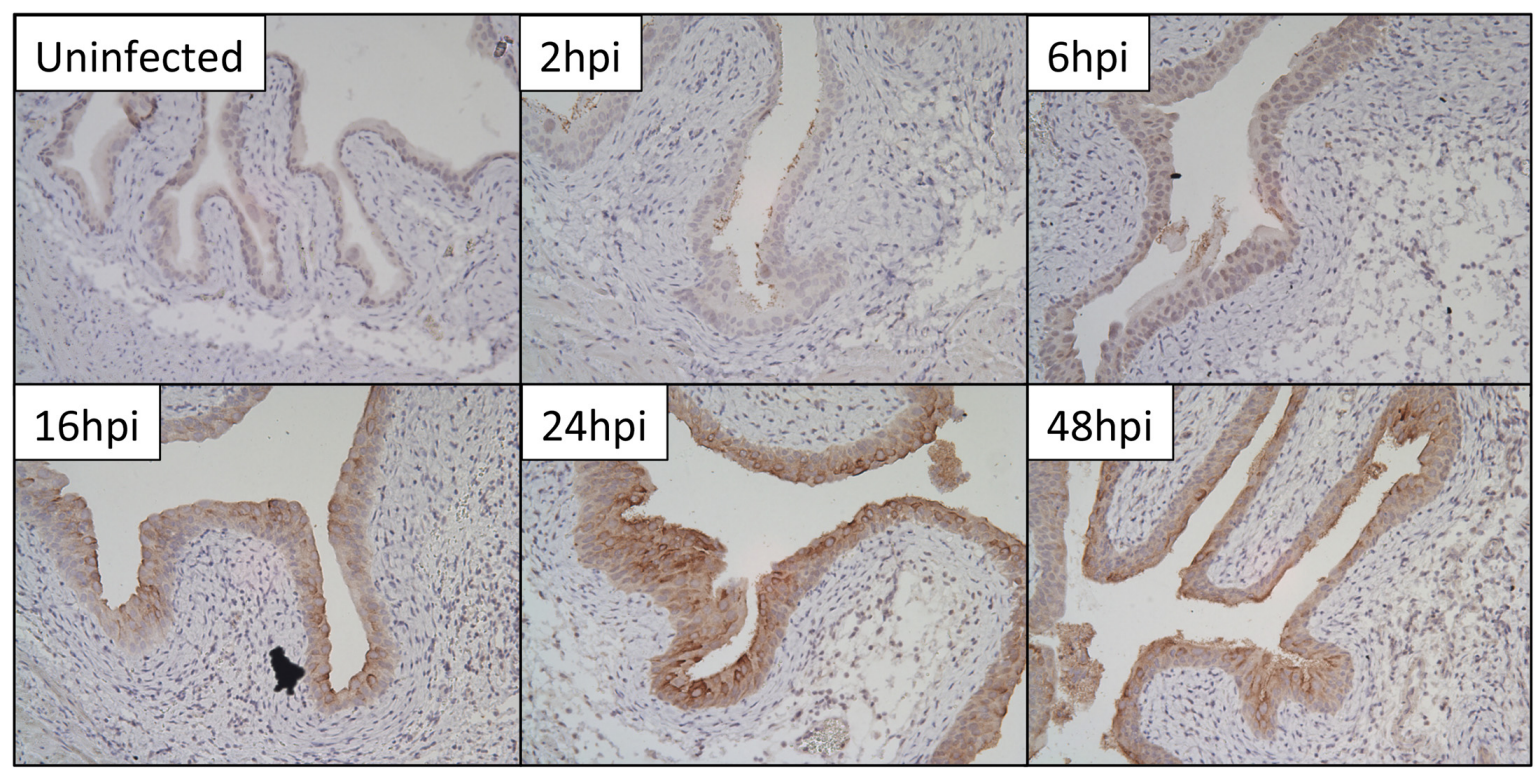
RegIIIγ protein localizes to urothelium following UPEC infection. RegIIIγ immunoreactivity is absent in uninfected bladders. Non-specific staining of UPEC is seen 2 and 6 hpi. Beginning 16 hpi, patchy urothelial immunoreactivity is observed. Urothelial RegIIIγ expression is most prominent 24 hpi and persists to a lesser extent 48 hpi. All figures are 20x magnification.

To localize RegIIIγ expression in bladders following UPEC infection, we performed immunostaining. RegIIIγ was undetectable in naïve bladders and at 2 and 6 hpi. Beginning 16 hpi we observed strong, patchy reactivity within urothelium by immunohistochemistry (**Fig 3**). Immunofluorescence microscopy confirmed cytoplasmic localization of RegIIIγ within urothelial cells, along with apical enrichment of RegIIIγ expression (**S2 Fig**). RegIIIγ immunoreactivity intensified 24 hpi, and remained detectable up to 72 hpi (**Fig 3 and S2 Fig**).

### RegIIIγ mRNA and protein expression during pyelonephritis

**Fig 4:**
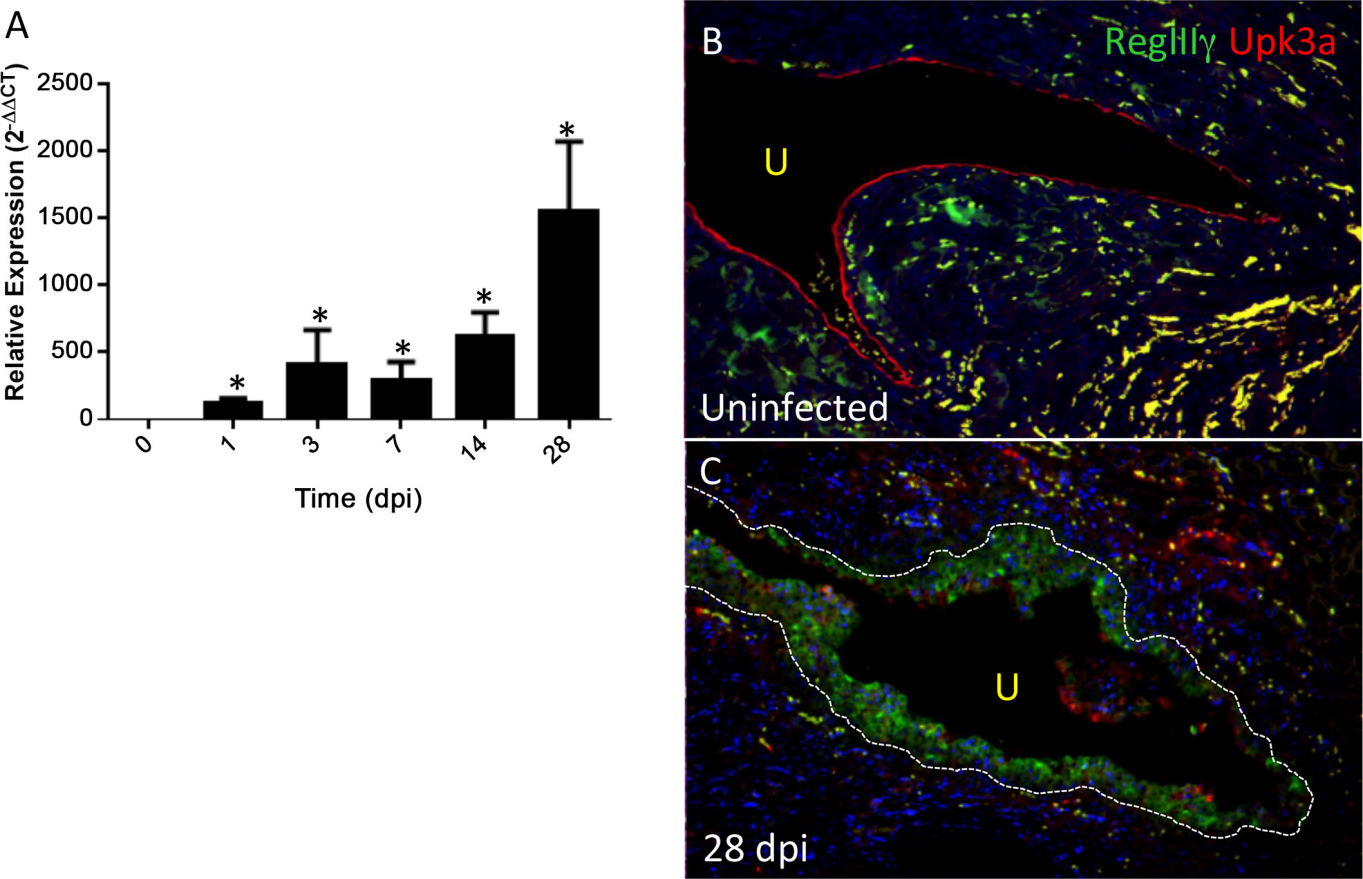
Induction of *RegIIIγ* mRNA and RegIIIγ protein by UPEC in C3H/HeOuJ kidneys. (**A**) Significant *RegIIIγ* mRNA induction by UPEC in C3H/HeOuJ kidneys, with progressive accumulation over time. (* P = 0.0286, Mann-Whitney U test versus uninfected C3H/HeOuJ kidneys, n = 4 kidneys/time). (**B**) Absent RegIIIγ immunostaining in uninfected C3H/HeOuJ urothelium, which exhibits uniform apical expression of Uroplakin 3a (Upk3a; red). U: Urinary space. (**C**) RegIIIγ protein (green) localizes to the hypertrophied urothelium of C3H/HeOuJ kidneys 28 dpi, with reduced expression of Upk3a. Urothelial border is indicated by dashed line. Both images are 20x magnification.

To evaluate the expression of *RegIIIγ* during pyelonephritis, we challenged C3H/HeOuJ mice with UPEC (strain CFT073). The C3H/HeOuJ strain is more susceptible to pyelonephritis following transurethral UPEC inoculation.[32,33,34] When challenged with 10^8^ CFU UPEC, C3H/HeOuJ kidneys developed persistent bacterial burden up to 28 days post infection (dpi; **S3 Fig**). Renal *RegIIIγ* mRNA levels significantly increase 1 dpi and continue to rise at later time points (14 and 28 dpi; **Fig 4A**). Uninfected C3H/HeOuJ renal urothelium exists as a monolayer with uniformly apical Uroplakin 3a (Upk3a) and absent RegIIIγ protein expression (**Fig 4B**). At 28 dpi, the renal urothelium appeared hyperplastic with reduced Upk3a expression and diffuse expression of RegIIIγ protein (**Fig 4C**).

### Urinary HIP/PAP levels increase during UTI

**Fig 5:**
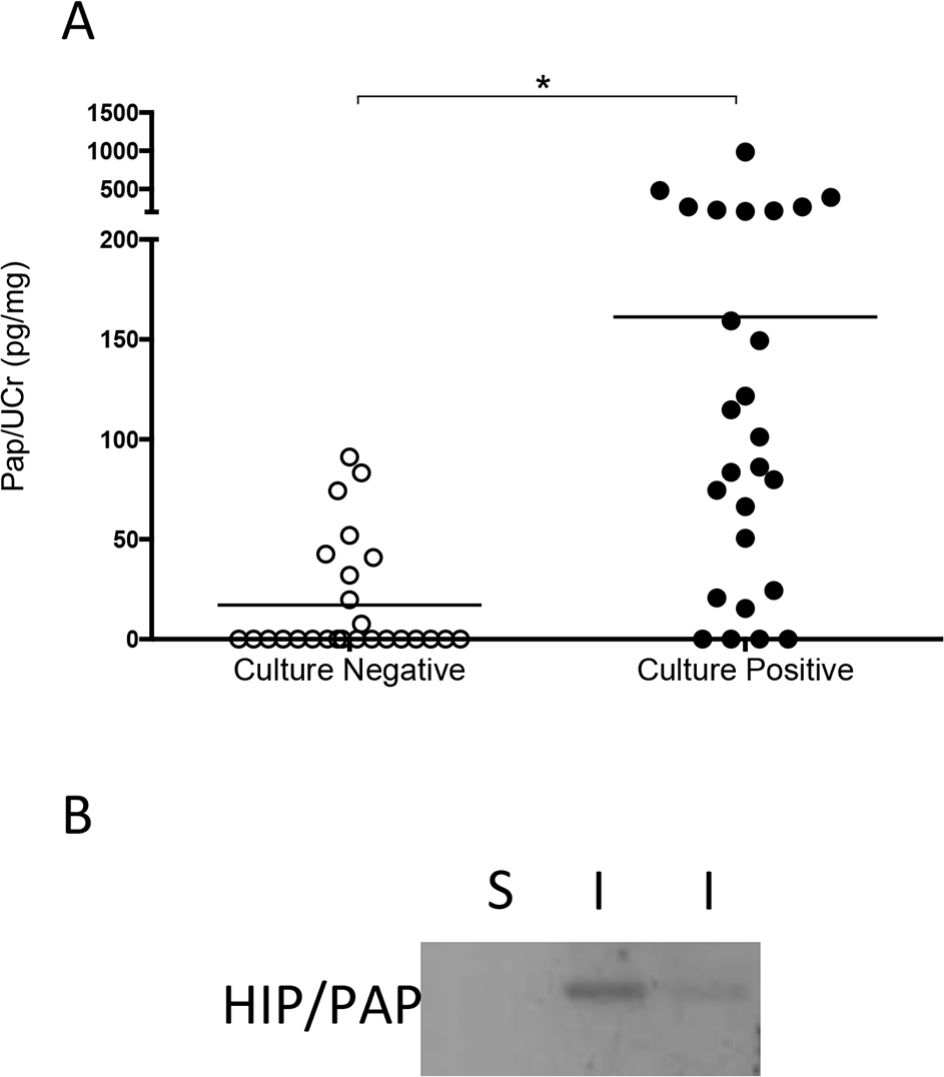
The human RegIIIγ orthologue, HIP/PAP, is secreted in infected human urine. (**A**) HIP/PAP levels were quantified by ELISA and adjusted for urine creatinine to control for differences in urine concentration. The resulting PAP/Cr ratio is significantly elevated in urine from patients with positive urine cultures, compared to sterile urine (n = 26 urines/group; * *P* < 0.0001, Mann-Whitney U test). The horizontal line indicates the mean. (**B**) Western blotting demonstrates absent of HIP/PAP in sterile (S) urine, whereas infected (I) urine from two children with UTI exhibits immunoreactivity.

In humans, the Hepatocarcinoma-Intestine-Pancreas / Pancreatitis Associated Protein (HIP/PAP) is orthologous to RegIIIγ and 67% identical at the amino acid level.(**S4 Fig**). ELISA analysis demonstrates that human urinary HIP/PAP concentrations significantly increase during *E*.*coli* UTI (**Fig 5A**). 26 patients with positive urine cultures had average urine PAP/Cr ratios of 161.2 pg/mg (95% confidence interval [CI], 77–245 pg/mg), compared to 26 culture-negative urines that exhibited PAP/Cr ratios of 17.05 (95% CI, 5–29 pg/mg); p < 0.0001, Mann-Whitney U test. Western blots also demonstrated higher HIP/PAP levels in infected urine (**Fig 5B**).

### Recombinant RegIIIγ protein does not kill UPEC in vitro, and RegIIIγ deficiency does not affect UTI susceptibility in vivo

**Fig 6:**
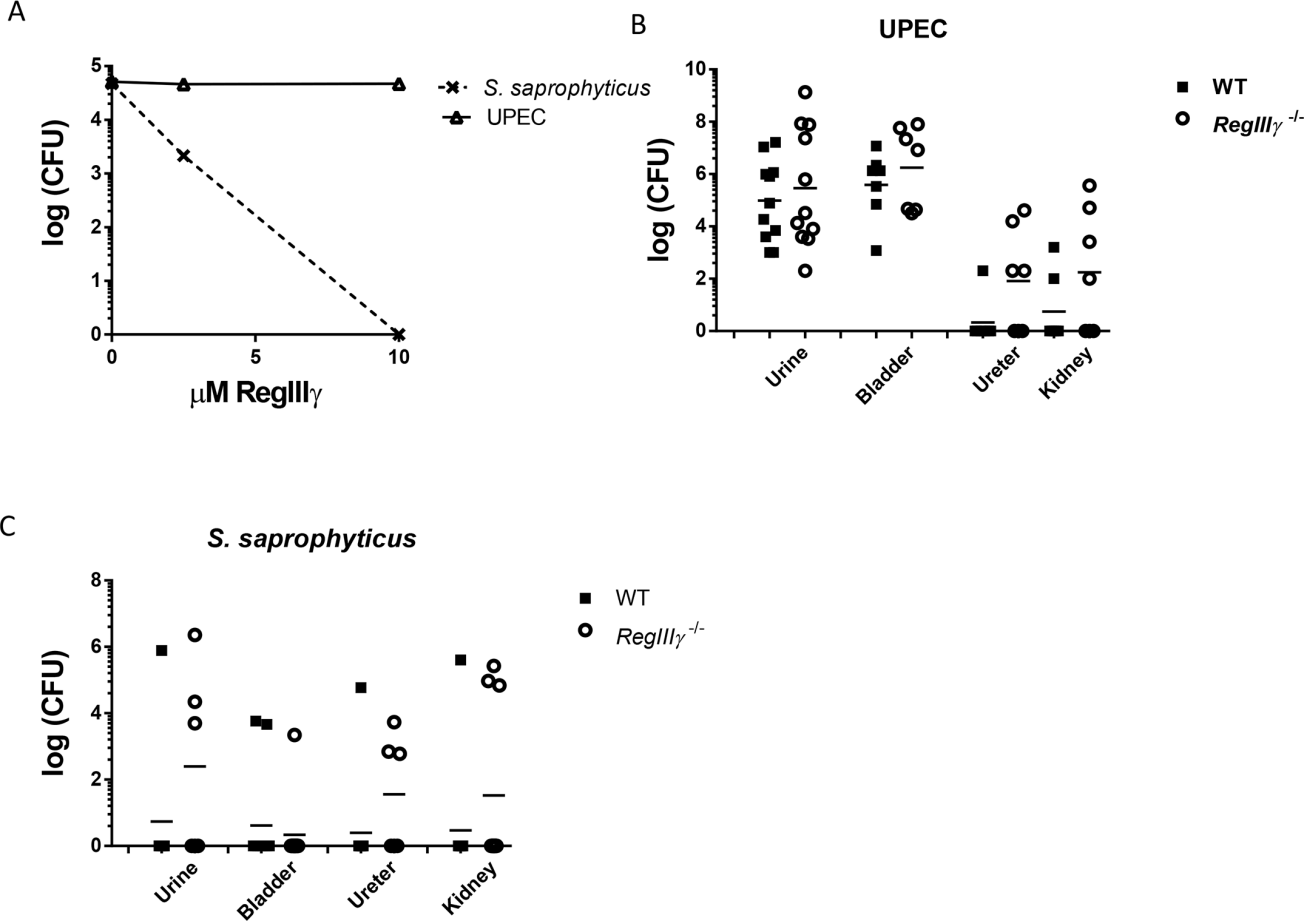
Recombinant RegIIIγ protein exhibits bactericidal activity toward *S*. *saprophyticus* but not UPEC, and *RegIIIγ* deletion does not affect susceptibility to experimental UTI. (**A**) Recombinant RegIIIγ kills *S*. *saprophyticus* not UPEC in a dose-dependent manner. 10^5^ CFU bacteria were incubated with indicated concentrations of recombinant C-terminally cleaved RegIIIγ for 2 hours at 37°C in 10 mM MES, pH 5.5 with 25 mM NaCl, and colonies were enumerated. (**B-C**) Experimental UTI. Comparable bacterial burden in wildtype (WT) and *RegIIIγ* ^*-/-*^ urinary tracts following inoculation with 10^8^ CFU of UPEC strain UTI89 (**B**) or *S*. *saprophyticus* (**C**). There was no significant difference between genotypes by Mann-Whitney U test (*p* > 0.05). Horizontal lines indicate geometric means.

To determine whether RegIIIγ displays bactericidal activity toward uropathogenic bacteria, we expressed recombinant (r)RegIIIγ protein in *E*. *coli* and purified it from bacterial inclusion bodies.[12,28,29] Antimicrobial kill assays show that rRegIIIγ kills *S*. *saprophyticus* in dose-dependent fashion, but does not exhibit bactericidal activity against UPEC (strain UTI89, **Fig 6A**).

To confirm these results in a model system, we evaluated the effects of *RegIIIγ* deletion on bacterial clearance. We confirmed absence of *RegIIIγ* mRNA and RegIIIγ protein in *RegIIIγ* ^*-/-*^ bladders by qRT-PCR, immunostaining, and immunoblotting following experimental UTI (**Fig 2C, S2 Fig, and data not shown**). We used an inoculum of 10^8^ CFU of UPEC strain UTI89, identical conditions to those in which we originally identified *RegIIIγ* mRNA expression, and investigated bacterial burden 24 hpi. We did not observe significant differences in urine, bladder, ureter, or kidney bacterial burden between *RegIIIγ* ^*-/-*^ and wildtype C57BL/6J control mice (**Fig 6B**). We also did not observe a difference in bacterial burden when the inoculum was reduced to 10^6^ or 10^7^ CFU, or when we evaluated bacterial burden 16, 24, 48, or 72 hpi (**S5 Fig**). Since RegIIIγ preferentially kills Gram-positive bacteria, we performed parallel experiments challenging *RegIIIγ* ^*-/-*^ and wild-type mice with 10^8^ CFU of uropathogenic *Staphylococcus saprophyticus* and *Enterococcus faecalis*. However, we did not observe any difference in susceptibility of *RegIIIγ* ^*-/-*^ mice to these Gram-positive bacteria 24 hpi (**Fig 6C and S6 Fig**).

## Discussion

### Bladder AMPs are identified on the basis of their transcriptional induction by UPEC

The purpose of this study was to identify AMPs within the bladder on the basis of their transcriptional induction during experimental UTI. This unbiased approach identified a set of AMPs with diverse mechanisms of antimicrobial activity. Most of these AMPs have been evaluated in the setting of UTI. Loss of function studies have established a role for Lcn2 production by bladder urothelium and α-intercalated cells of renal collecting ducts in response to UPEC, achieved in part through iron scavenging.[35,36] Peptide fragments from human lactoferrin exhibit bactericidal activity toward UPEC *in vitro* and when administrated orally accumulate in urine and reduce bladder and kidney colonization by UPEC *in vivo*.[37] S100a8 and S100a9 heterodimerize to form calprotectin, a neutrophil derived AMP with bacteriostatic activity due to chelation of micronutrient metals such as zinc and manganese.[38] Previous studies have demonstrated accumulation of neutrophil-derived S100a8 and S100a9 proteins during experimental UTI, but *S100a9* deletion does not confer increased susceptibility to infection.[39] Phagocytes express RNase6 protein during human and murine UTI, and recombinant human and murine RNase6 proteins are bactericidal toward UPEC *in vitro*.[23] Along with these AMPs, here we identified *RegIIIβ* and *RegIIIγ* as inducible AMPs during experimental UTI. This project focused on RegIIIγ due to its high magnitude of induction.

### RegIIIγ protein expression increases during UTI and localizes to urothelial cells

We confirmed induction of RegIIIγ protein in infected mouse urine and bladders by immunoblotting and localized RegIIIγ protein to infected bladder urothelium. Our study adds urothelium to the list of mucosal surfaces capable of RegIIIγ expression. Previous studies have identified constitutive *RegIIIγ* mRNA expression in intestinal epithelium and localized RegIIIγ protein to mucus coating these cells.[14,15] In addition to this “homeostatic” pool of RegIIIγ, mucosal injury leads to cytokine-mediated induction of RegIIIγ production.[40,41] RegIIIγ mRNA expression also occurs in developing airway epithelium as well as in lung epithelium during *S*. *aureus* pneumonia.[12,42,43] We were unable to detect RegIIIγ protein in bladder extracts or localize RegIIIγ protein to bladder urothelium in the absence of infection, but RegIIIγ mRNA was readily detected under these conditions with higher levels in bladder than other urinary tract organs. This suggests separate constitutive and inducible pools of RegIIIγ mRNA, which are likely subject to differential regulation.

### Potential mechanisms of *RegIIIγ* mRNA regulation during UTI

While the mechanisms responsible for urothelial expression of RegIIIγ mRNA are not elucidated in our study, certain inferences can be made based on published findings outside of the urinary tract. Homeostatic RegIIIγ mRNA expression in intestine depends on Toll-like receptor (Tlr) 4 signaling in epithelial cells through the adaptor proteins Myd88 and Trif.[15,17,41,44] Intestinal RegIIIγ mRNA expression is significantly impaired in germ-free mice, and bacterial colonization or administration of bacterial products such as lipopolysaccharide (LPS) or flagellin induces RegIIIγ expression via Tlr4 and Tlr5, respectively.[13,16,45] These studies raise the question of whether RegIIIγ induction by UPEC is dependent on LPS, flagellin, or a different bacterial product in the urinary tract.

Previous transcriptome studies have identified RegIIIγ mRNA induction during cystitis and offer clues about its regulation by bacteria.[46,47,48,49] UPEC lacking the FimH adhesin elicit 9.5-fold less RegIIIγ mRNA induction than the parental strain, attesting to a potential role in FimH interactions with uroplakin plaque covering the urothelium in RegIIIγ expression.[47,50] However, RegIIIγ mRNA expression does not uniquely depend on Gram-negative bacteria, since intravesical inoculation of *Streptococcus agalactiae* results in 8.7-fold higher bladder RegIIIγ mRNA levels versus uninfected controls.[46] Thus, multiple pathogen associated molecular patterns (PAMPs) may be responsible for bladder RegIIIγ expression, just as LPS and flagellin regulate RegIIIγ mRNA levels in the gut.[16,45]

Alternatively, induction of RegIIIγ mRNA in UPEC infected bladders may occur as consequence of cytokine signaling. In the lung, interleukin-6 (IL-6) leads to phosphorylation of Signal transducer and activator of transcription (Stat) 3, which translocates to the nucleus and mediates RegIIIγ mRNA transcription.[12,51] In the skin of mice and humans with psoriasis, HIP/PAP and RegIIIγ undergo robust mRNA and protein induction under the direction of IL-17, another cytokine capable of eliciting Stat3 phosphorylation.[11] In the stomach, the *Helicobacter pylori* CagA protein elicits IL-11 production, which leads to Stat3 phosphorylation and RegIIIγ expression.[52] In the mouse colon, infectious and chemical agents trigger production of IL-22, which induces epithelial RegIIIγ expression.[40,53,54] Altogether, these observations warrant future studies of Stat3 and its upstream regulatory cytokines in the regulation of RegIIIγ during experimental UTI.

### Urinary RegIIIγ and HIP/PAP are markers of urothelial injury

In this study, we demonstrate that RegIIIγ and HIP/PAP proteins accumulate in the urine of mice and humans with UTI. Previous studies in rats and humans have identified induction of RegIIIγ orthologues in the context of hemorrhagic cystitis, bladder cancer, and interstitial cystitis.[55,56,57] In rats, bladder *PAP-III* mRNA levels increase following cyclophosphamide treatment, and PAP-III protein uniquely localizes to the umbrella cells of bladders with hemorrhagic cystitis.[57] In humans with bladder cancer, HIP/PAP is detected in tumors by immunoblotting and urine HIP/PAP levels rise.[56] Patients with bladder cancer and IC exhibit elevated urine concentrations of HIP/PAP protein.[55,56] Taken together, these studies implicate RegIIIγ and HIP/PAP as markers of urothelial injury. It will be insightful to determine the clinical and diagnostic value of urine HIP/PAP levels in patients with urothelial injury, including UTI and obstructive uropathy.

### *RegIIIγ* deletion does not confer susceptibility to experimental UTI

It is perhaps not surprising that genetic ablation of RegIIIγ did not affect host susceptibility to UPEC UTI, since recombinant RegIIIγ did not exert direct bactericidal activity toward UPEC. The antimicrobial activity of both RegIIIγ and HIP/PAP requires binding to peptidoglycan, which is inaccessible in the Gram-negative cell wall.[13] Moreover, LPS inhibits multimerization of HIP/PAP and presumably RegIIIγ, a required step for their membrane insertion into Gram-positive target cells.[58] Nonetheless, there is experimental precedent that RegIIIγ promotes resolution of Gram-negative infections *in vivo*. Recombinant RegIIIγ and HIP/PAP proteins prevent lethal *Citrobacter* colitis.[40] Moreover, genetic deletion of RegIIIγ increases susceptibility to intestinal infection by *Salmonella enteritidis*, a Gram-negative bacterium.[14] Thus, while it is unlikely that RegIIIγ or HIP/PAP exhibit direct microbicidal activity, it remains conceivable that they may work cooperatively with other AMPs or host immune cells to serve an antimicrobial function in certain Gram-negative infections.

### Potential roles for RegIIIγ and HIP/PAP in the urinary tract

While our findings argue against a direct antimicrobial role for urothelial RegIIIγ during UTI caused by UPEC, alternative roles for RegIIIγ merit consideration. In skin, RegIIIγ and HIP/PAP promote keratinocyte proliferation and prevent terminal differentiation in states of wound repair and psoriasis.[11] Bladder urothelial cells exfoliate following UPEC exposure, prompting a wave of progenitor cell proliferation and differentiation that regenerates the mucosal layer.[59,60] Based on its function in skin and expression in urothelium, RegIIIγ may regulate urothelial cell proliferation and/or differentiation during UTI. Alternatively, local expression of RegIIIγ within the urothelium may serve immumodulatory roles. In rats, the RegIII family member, PAP-I, promotes Stat3 phosphorylation in macrophages, blocking NF-κB activation and inhibiting the inflammatory response.[61] This raises the question of whether *RegIIIγ* ^*-/-*^ mice exhibit alterations in Stat3 activation and increased inflammation following experimental UTI. In support of this concept, bacteria directly interact with the epithelial surface in *RegIIIγ* ^-/-^ intestine, leading to immune cell activation.[14,15]

## Conclusions

UPEC infection leads to induction of RegIIIγ mRNA and RegIIIγ protein, which localizes to bladder and renal urothelial cells and is secreted in infected urine. RegIIIγ and HIP/PAP represent novel biomarkers of human and murine UTI, respectively. The biological significance of these proteins in the infected urinary tract remains unknown.

## Supporting Information

S1 Fig *RegIIIγ* mRNA levels are significantly higher in uninfected bladder, compared to ureter and kidney (* *P* = 0.0286, Mann-Whitney U test,n = 4 organs/group). (TIF)

S2 Fig RegIIIγ expression by bladder urothelial cells following UPEC inoculation. Immunofluorescence microscopy localizes RegIIIγ (green) to apical projections of wild-type (WT) urothelial cell layers 24 and 72 hpi. Simultaneously stained *RegIIIγ* ^-/-^ sections exhibit non-specific staining at these times post infection. Nuclei are identified with DAPI (blue). Dashed lines demarcate the urothelial border. U: Urinary space. All images are 40x magnification.
(TIF)

S3 Fig Persistent UPEC colonization of C3H/HeOuJ kidneys. Female C3H/HeOuJ mice with transurethrally inoculated with UPEC strain CFT073 (10^8^ CFU). Dpi: days post infection. Squares indicate individual kidneys. The horizontal line indicates the geometric mean.
(TIF)

S4 Fig Human HIP/PAP is 67% identical in amino acid sequence to mouse RegIIIγ. Identical amino acid residues are highlighted in black.
(TIF)

S5 Fig Effect of varying infection duration and magnitude of UPEC inoculum on urinary tract bacterial burden in *RegIIIγ* ^*-/-*^ versus WT mice. (A) No significant difference in bladder or kidney bacterial burden was observed when WT and *RegIIIγ* ^*-/-*^ mice were challenged with 10^7^ CFU of UTI89 and sacrificed at 16, 24, 48, or 72 hpi (*p* > 0.05, Mann-Whitney U test). (B) No significant difference in urinary tract bacterial burden was noted when WT and *RegIIIγ* ^*-/-*^ mice were challenged with 10^6^ CFU of UTI89 and sacrificed 24 hpi (*p* > 0.05, Mann-Whitney U test).
(TIF)

S6 Fig Comparable bacterial burden in wildtype (WT) and *RegIIIγ* ^*-/-*^ urinary tracts following inoculation with 10^8^ CFU of E. faecalis strain 0852. There was no significant difference between genotypes by Mann-Whitney U test (*p* > 0.05). Horizontal lines indicate geometric means.
(TIF)

S1 Table QRT-PCR primers. (XLSX)

S2 Table *Mus musculus* genes encoding known or putative antimicrobial peptides [6,27,28]. (XLSX)
