# Supplementary figures and images for: Expression and Significance of the HIP/PAP and RegIIIγ Antimicrobial Peptides during Mammalian Urinary Tract Infection
Source: PLoS One. 2015 Dec 10;10(12):e0144024. doi: 10.1371/journal.pone.0144024 (PMC4675559; doi:10.1371/journal.pone.0144024)

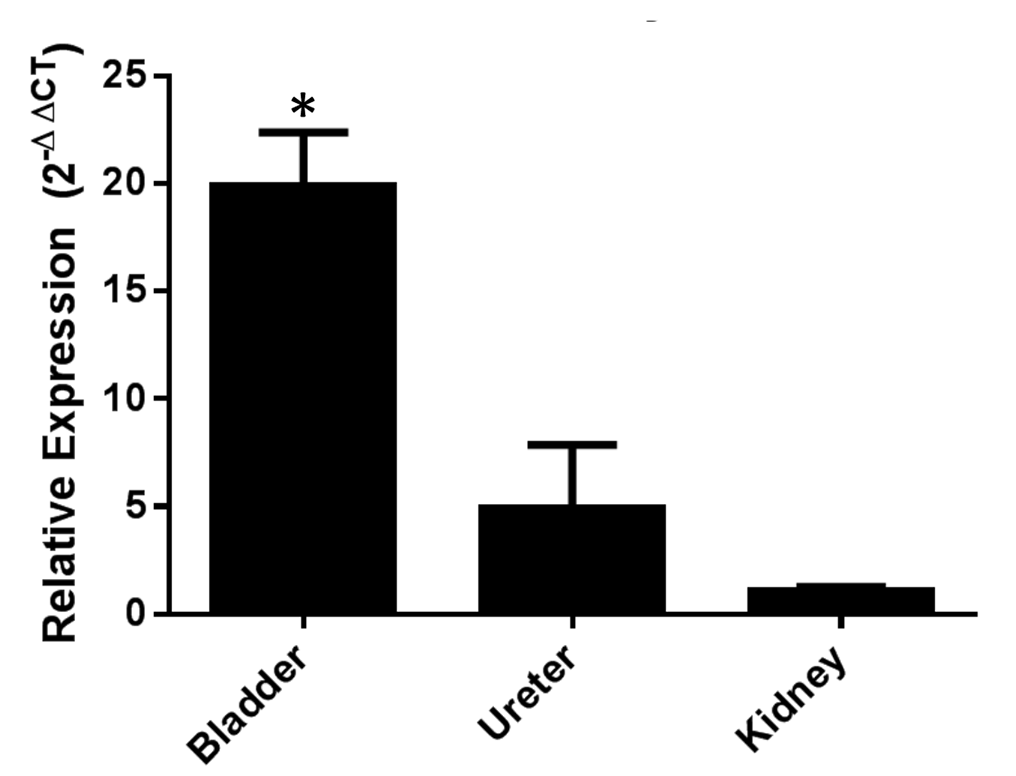

Supplement: S1 Fig — (TIF) [file pone.0144024.s001.tif]

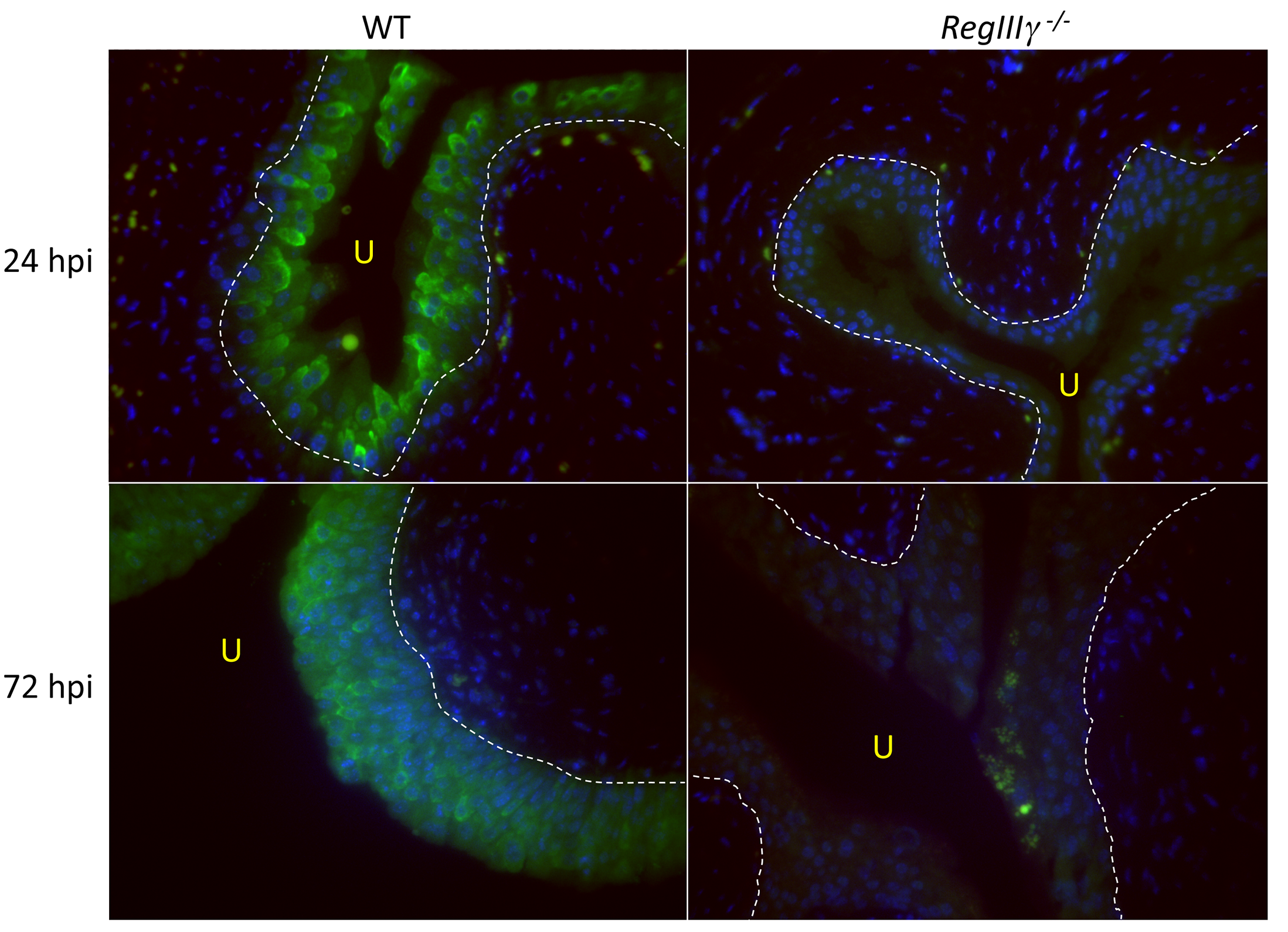

Supplement: S2 Fig — Immunofluorescence microscopy localizes RegIIIγ (green) to apical projections of wild-type (WT) urothelial cell layers 24 and 72 hpi. Simultaneously stained RegIIIγ -/- sections exhibit non-specific staining at these times post infection. Nuclei are identified with DAPI (blue). Dashed lines demarcate the urothelial border. U: Urinary space. All images are 40x magnification. (TIF) [file pone.0144024.s002.tif]

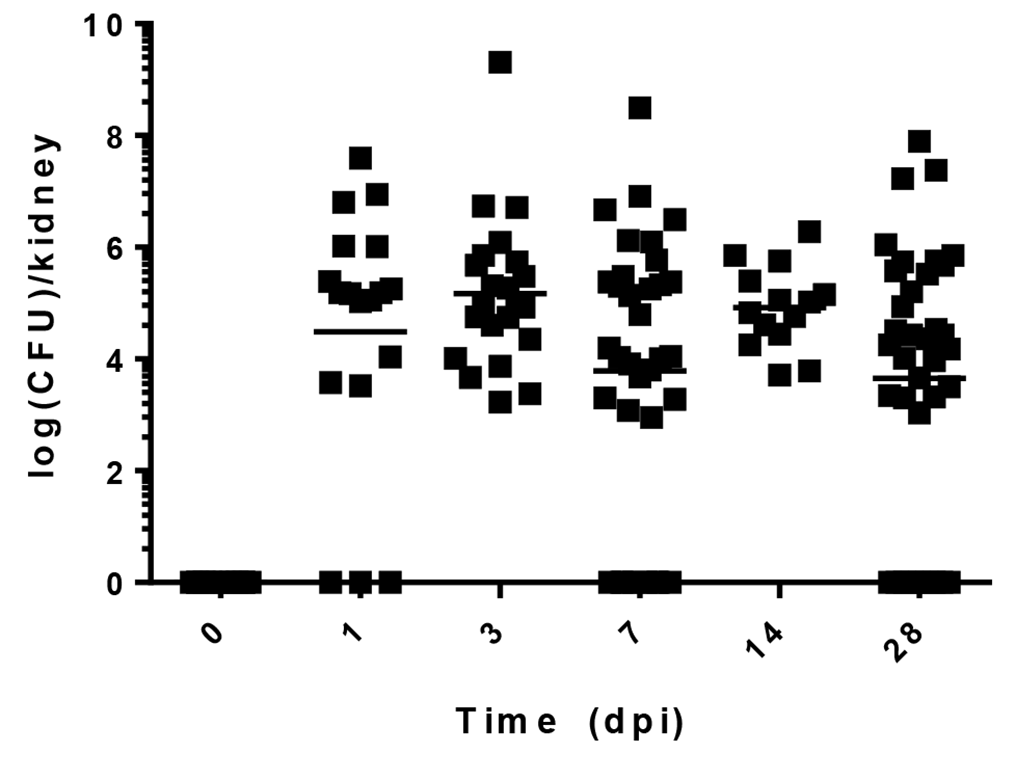

Supplement: S3 Fig — Female C3H/HeOuJ mice with transurethrally inoculated with UPEC strain CFT073 (108 CFU). Dpi: days post infection. Squares indicate individual kidneys. The horizontal line indicates the geometric mean. (TIF) [file pone.0144024.s003.tif]

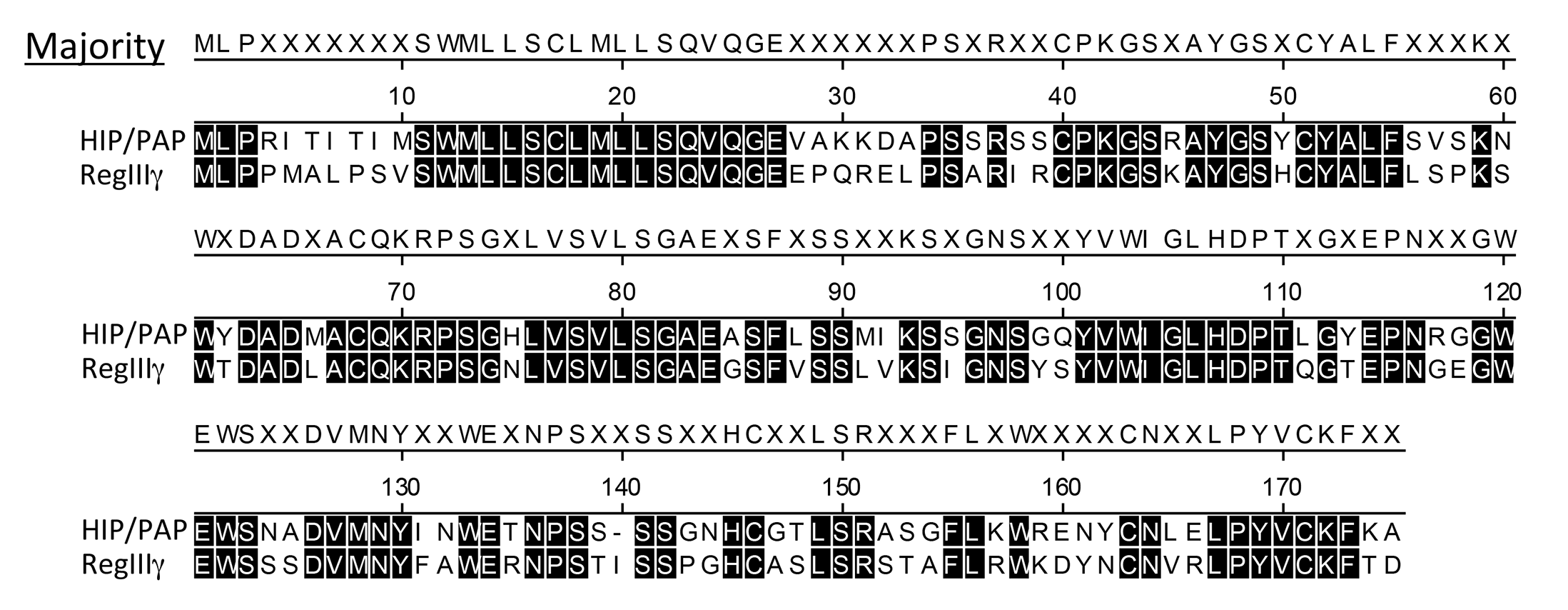

Supplement: S4 Fig — Identical amino acid residues are highlighted in black. (TIF) [file pone.0144024.s004.tif]

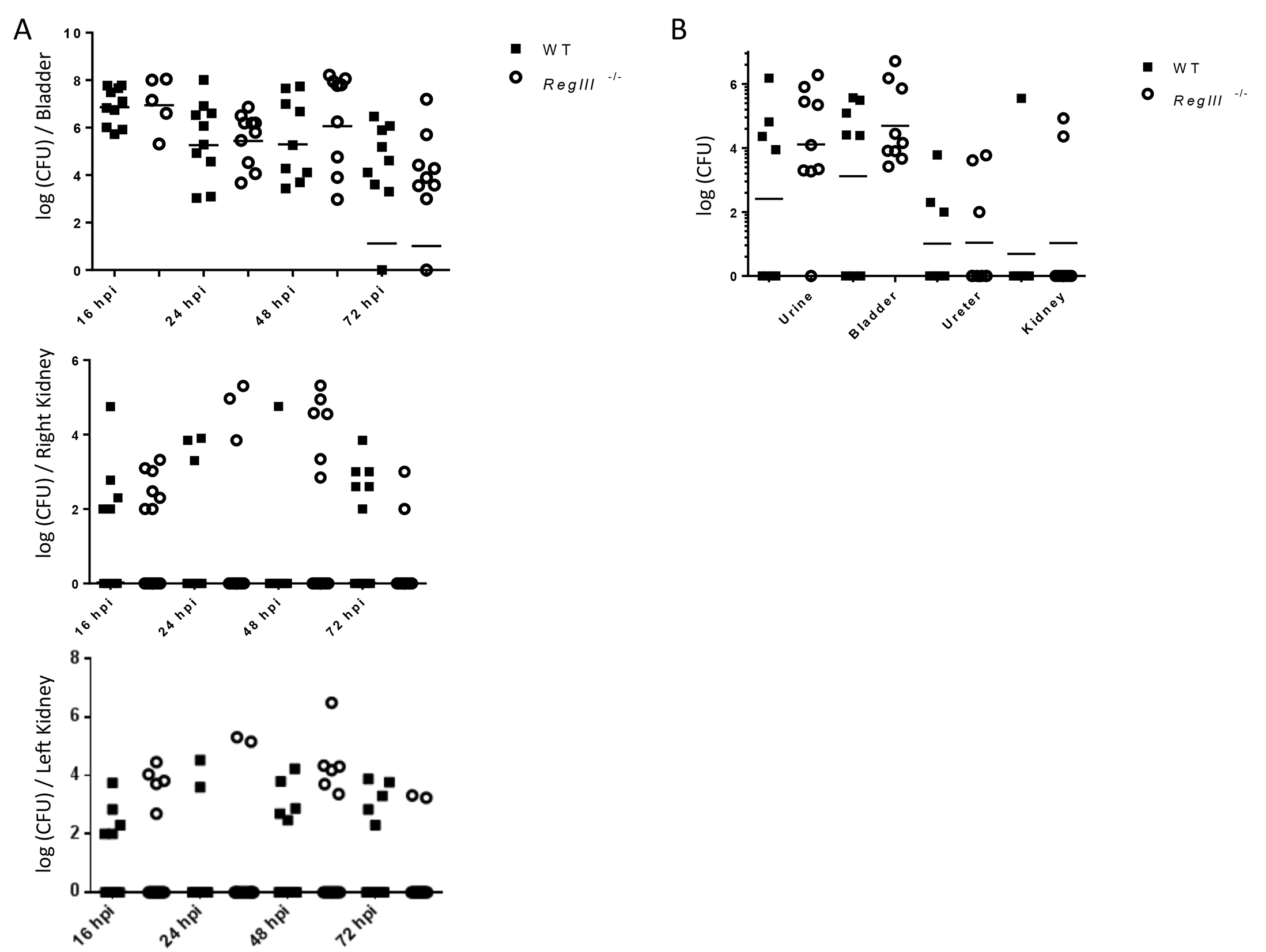

Supplement: S5 Fig — (A) No significant difference in bladder or kidney bacterial burden was observed when WT and RegIIIγ -/- mice were challenged with 107 CFU of UTI89 and sacrificed at 16, 24, 48, or 72 hpi (p > 0.05, Mann-Whitney U test). (B) No significant difference in urinary tract bacterial burden was noted when WT and RegIIIγ -/- mice were challenged with 106 CFU of UTI89 and sacrificed 24 hpi (p > 0.05, Mann-Whitney U test). (TIF) [file pone.0144024.s005.tif]

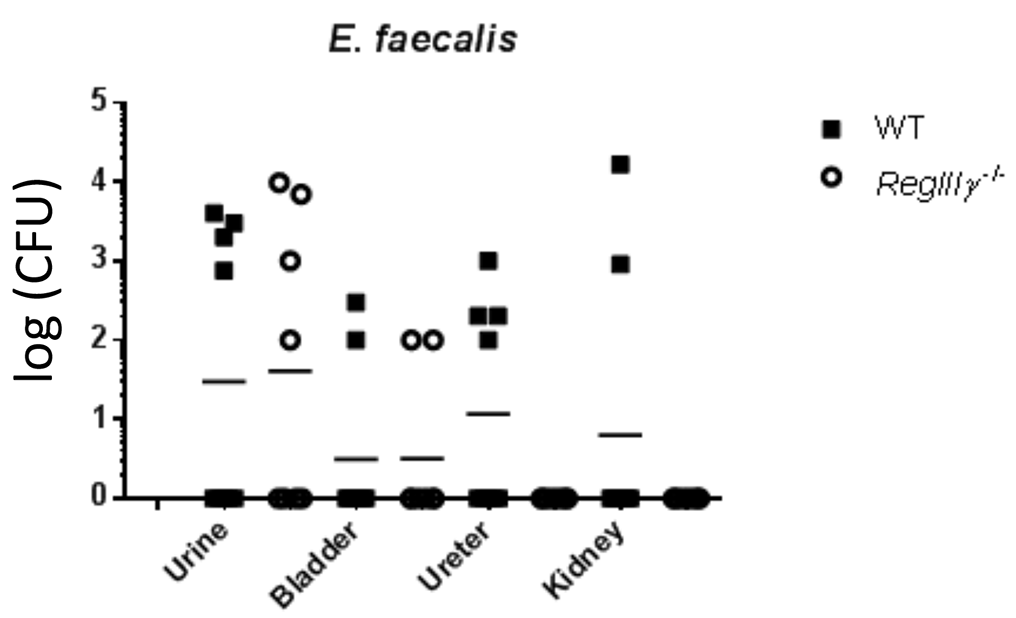

Supplement: S6 Fig — There was no significant difference between genotypes by Mann-Whitney U test (p > 0.05). Horizontal lines indicate geometric means. (TIF) [file pone.0144024.s006.tif]
